# Who Benefits from Fermented Food Consumption? A Comparative Analysis between Psychiatrically Ill and Psychiatrically Healthy Medical Students

**DOI:** 10.3390/ijerph19073861

**Published:** 2022-03-24

**Authors:** Michał Seweryn Karbownik, Łukasz Mokros, Edward Kowalczyk

**Affiliations:** 1 Department of Pharmacology and Toxicology, Medical University of Lodz, Żeligowskiego 7/9, 90-752 Lodz, Poland; edward.kowalczyk@umed.lodz.pl; 2 Department of Clinical Pharmacology, Medical University of Lodz, Kopcińskiego 22, 90-153 Lodz, Poland; lukasz.mokros@umed.lodz.pl

**Keywords:** probiotic, fermented food, depression, anxiety, psychiatric patients, psychiatrically healthy, medical students, extent of association

## Abstract

Probiotic therapies and fermented food diets hold promise for improving mental health. Although in this regard psychiatric patients appear to benefit more than healthy individuals, no research has been performed to directly evaluate this hypothesis. The present study examined a cohort of medical students facing a stressful event, and some of the students reported suffering from chronic psychiatric diseases. The amount of fermented food consumption was calculated with the use of seven-day dietary records, while depressive and anxiety symptoms were assessed with the use of the Patient Health Questionnaire-9 and Generalized Anxiety Disorder-7, respectively. In psychiatrically healthy medical students under psychological stress (*n* = 372), higher fermented food consumption was associated with more depressive and anxiety symptoms. In contrast, psychiatrically ill medical students (*n* = 25, 6.3% of all the participants) were found to present a negative association between the amount of fermented food consumed and the severity of depressive symptoms (adjusted β −0.52, 95% CI −0.85 to −0.19, *p* = 0.0042); however, this relationship was insignificant for anxiety symptoms (adjusted β −0.22, 95% CI −0.59 to 0.15, *p* = 0.22). A significant interaction was found between the consumption of fermented food and psychiatric diagnosis in predicting depressive symptoms (*p* = 0.0001), and a borderline significant interaction for anxiety symptoms (*p* = 0.053). In conclusion, psychiatrically ill people, but not healthy ones, may benefit from fermented food consumption in terms of alleviation of depressive symptoms. Our findings require cautious interpretation and further investigation.

## 1. Introduction

The human digestive tract is full of live microbes. They are not passive space fillers, but constitute a symbiotic community indispensable to human health. This gut microbiota offers multiple benefits to the host, such as production of nutrients, defense against pathogens, and regulation of immune functioning [1]. The microbes are also believed to interact with the brain by participating in the so-called gut–brain axis (GBA). Through this bidirectional communication, microorganisms may influence the brain structure and function, and ultimately our emotions and behaviors [2,3]. It has been proposed that microbes within the GBA act by secretion of cytokines, modulation of transmission through the vagus nerve, and action of microbiota-derived neurotransmitters, their precursors, or other metabolites that may regulate intermediate pathways or directly affect the brain [2,3,4,5]. For example, short-chain fatty acids (SCFAs), the main gut microbiota metabolites, release neurotransmitters and gut hormones from enteroendocrine cells, and stimulate vagal pathways. SCFAs also penetrate the blood–brain barrier to alleviate central neuroinflammatory processes and stimulate neurotrophic factors [4]. Another example is the microbiota-mediated tryptophan metabolism that leads to indole derivatives which appear to exert anti-inflammatory effects in the brain [5].

Pharmacological or dietary manipulations within the gut microbiota may substantially modulate GBA signaling [2]. Consequently, gut microbiota-targeted therapies and dietary interventions hold promise for an adjunctive treatment of psychiatric diseases and improving the mental well-being of healthy people [2,3,4,5]. Several recent systematic reviews of human controlled trials with meta-analyses have suggested some improvement in depressive and anxiety symptoms following the intake of probiotic preparations [6,7,8,9,10,11,12,13]. Similarly, an association between higher consumption of fermented food and improvement in mental health indices has been noted in some observational studies [14,15,16]. It appears that patients suffering from clinical depression benefit from gut microbiota-based therapies or diets more than healthy individuals or non-psychiatric patients do [8,9,10,11]. However, this presumption was made solely on the comparison of individual studies, and no research has so far directly evaluated the difference in effect size between psychiatric patients and mentally healthy individuals.

The aim of the present study was to compare psychiatrically ill medical students and their psychiatrically healthy schoolmates with regard to the extent of the relationship between fermented food consumption and depressive and anxiety symptoms experienced under psychological stress.

## 2. Materials and Methods

### 2.1. Study Design and Ethical Considerations

In our previous study, the association between fermented food consumption and depressive and anxiety symptoms has been tested among psychiatrically healthy medical students under psychological stress [17]. In order to minimize observer bias, that study had no specific exclusion criteria and psychiatrically ill volunteers could also participate, but their records were not analyzed as per the pre-specified study protocol [18]. In contrast, the present study compares data from psychiatrically ill medical students with that of psychiatrically healthy ones. It provides a direct comparison of psychiatrically diagnosed and psychiatrically healthy individuals, as both groups constitute a coherent cohort of medical students.

The study was approved by the Bioethics Committee of the Medical University of Lodz, Poland (RNN/111/20/KE). All participants were required to provide electronic informed consent. The study was survey-based. After providing baseline sociodemographic, psychological, and health-related data, the participants performed a seven-day dietary record. On the last day, mental health symptoms were evaluated, and a day later, the final subject exam was carried out as a psychological stressor.

### 2.2. Study Participants

Third-year medical students at the Medical University of Lodz, Poland, academic year 2019/2020, were invited to participate. The cohort was comprised of psychiatrically healthy and psychiatrically ill individuals. The only exclusion criterion was a formal inability to sit the first attempt of the final examination in Pharmacology (the model of stress). However, after study completion, the records of participants who declared taking systemic antimicrobial agents or being hospitalized (less than 30 days before the start or during the study) were excluded from the analysis, as were students taking psychotropic drugs without an underlying psychiatric diagnosis. The latter could indicate individuals requiring temporal psychiatric support, not reflecting typical psychiatric pathology.

### 2.3. Study Procedure and Research Instruments

At baseline (several days before the food recording), participants self-reported sociodemographic (year of birth, sex, socioeconomic status, and number of inhabitants in a place of family residence), anthropometric (body weight and height to calculate the body-mass index, BMI), and morbidity data. The fact of suffering from any chronic psychiatric disease was self-reported (“yes” or “no”), and participants were not asked to further detail the type of psychiatric condition if present. The other collected baseline characteristics included personality traits (assessed with the Polish version of the Big Five Inventory-Short, BFI-S [19,20]), the overall quality of diet (Polish version of the Starting the Conversation scale, STC [21,22]), mode of eating, physical activity (a measure partially validated [22] against the Polish version of the International Physical Activity Questionnaire Short form [23,24]), and cigarette smoking/use. In the same survey, at baseline, participants were questioned about their depressive symptoms (Polish version of the Patient Health Questionnaire-9, PHQ-9 scale [25,26]) and anxiety symptoms (Polish version of the Generalized Anxiety Disorder-7, GAD-7 scale [27,28]). PHQ-9 and GAD-7 assess mental health symptoms based on nine- and seven-symptom lists, respectively, and the participants indicate the frequency of experiencing each of the symptoms within the previous two weeks with a four-point Likert scale.

Seven days before the final examination in Pharmacology (the model of stress), the participants started recording their diet. They were provided with an illustrated electronic open-ended dietary record form (Google Forms) itemizing 34 food categories to repeatedly self-report the mass (grams) of foodstuffs consumed in a particular meal/beverage for seven days. Each time participants visually estimated the food mass, it was based on several photographs of different food portions or household measures that were depicted in the dietary record form with their masses in grams, as set according to a web-based dietary service, Ilewazy.pl (Edipresse Polska; Warsaw, Poland). Six out of the thirty-four examined food categories concerned fermented products: (1) cheese, (2) yogurt, kefir, and soured milk, (3) kvass and unpasteurized beer, (4) pickled cucumber and pickling juice, (5) sauerkraut and pickling juice, and (6) other fermented vegetables and their pickling juice (for details see Supplementary Material S1). The total seven-day mass of all consumed fermented foods was calculated for each participant.

On the last day of dietary recording and a day before the stressful final exam, depressive and anxiety symptoms were again evaluated with the use of PHQ-9 and GAD-7 scales. The instructions for the tools were modified to assess the symptoms experienced over the seven pre-exam days only, and such modifications have previously been applied [29,30]. Pre-exam PHQ-9 and GAD-7 scores were used as the outcome variables in the analyses. In the same survey, the participants were also questioned about their pre-exam quality of diet (STC scale), mode of eating, and physical activity. Additionally, the seven-day use of probiotic dietary supplements or medicinal products was recalled in that survey. One probiotic dose was considered equivalent to 100 g of fermented food [31,32]. Fermented food equivalents of probiotic preparations were added to the total mass of six fermented food categories consumed throughout the seven days of dietary recording [17]—this total measure constituted the predictor in the analyses.

Details regarding the study procedure and research instruments are provided in our primary study report [17].

### 2.4. Data Analysis

The characteristics of the two groups of participants (i.e., those diagnosed with psychiatric disorders and those who were not) were compared with the Mann–Whitney *U* test (continuous and ordinal variables) and Pearson’s χ^2^ test or Fisher’s exact test (categorical variables). The change in parameters of the subjects from baseline to pre-exam was evaluated with the Wilcoxon signed-rank test. Due to the need to additionally adjust for potential confounders and interaction testing, the association between the consumption of fermented food and mental health outcomes was examined with the use of parametric techniques (general linear modeling, GLM). However, a corresponding non-parametric analysis (Spearman’s rank correlation) was performed when applicable. Potential confounders were identified based on the significance of their associations with a predictor or outcome variable in univariate analyses (in psychiatrically ill or healthy participants). The following potential confounders were included in the analysis: sex, BMI, four personality traits (neuroticism, extraversion, agreeableness, and consciousness), pre-exam physical activity, and diet quality. Several sensitivity analyses were carried out to confirm the reported findings. *p*-values below 0.05 were considered statistically significant. The analyses were performed using STATISTICA Software version 13.3 (Statsoft; Tulsa, OK, USA). The underlying raw data have been made publicly available through the Mendeley Data repository (http://dx.doi.org/10.17632/kdxv4k6f5c.2 accessed on 24 March 2022).

## 3. Results

### 3.1. Basal Charactersitics of the Study Participants

In total, 397 medical students who met inclusion/exclusion criteria completed the study. Among them, 25 (6.3%) reported suffering from chronic psychiatric conditions. Among the psychiatrically ill participants, the mean age of the group was reported to be 22.5 years, with a little less than one-fourths being male. This group demonstrated similar characteristics to the psychiatrically healthy group (*n* = 372) apart from personality traits and mental health symptoms: psychiatrically diagnosed students reported a more neurotic personality and more severe depressive and anxiety symptoms at baseline. Twenty-four people (96%) presented at least mild depressive symptoms (PHQ-9 score ≥ 5 [25]) and twenty-one people (84%) reported at least mild anxiety symptoms (GAD-7 score ≥ 5 [27]). Detailed characteristics of psychiatrically ill medical students in relation to their healthy schoolmates are presented in Table 1.

### 3.2. Pre-Exam Characteristics of Psychiatrically Ill Medical Students

Similar to the healthy students [17], psychiatrically ill participants decreased their physical activity in the 7-day pre-exam period (*p* = 0.0076). Their overall quality of diet did not significantly change (*p* = 0.89), but they reported reduced consumption of margarine, butter, or meat fat (*p* = 0.0077). Their depressive and anxiety symptoms did not significantly alter in the pre-exam period in comparison to the baseline level (*p* = 0.28 and *p* = 0.22, respectively). The psychiatrically ill group presented similar scores in the final subject exam as the healthy group (*p* = 0.83). They consumed a median of 647.5 g (1st–3rd quartiles 184.8 to 1187.2) of fermented food in the 7-day pre-exam period, which was not much different from that of the healthy group (*p* = 0.75).

### 3.3. Consumption of Fermented Food in Relation to Depressive and Anxiety Symptoms

**Figure 1:**
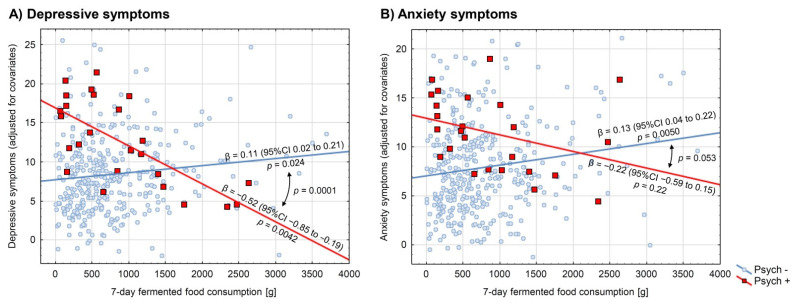
Association between consumption of fermented food and (**A**) depressive and (**B**) anxiety symptoms in psychiatrically ill medical students and their psychiatrically healthy schoolmates. Depressive symptoms were evaluated with the use of the Patient Health Questionnaire-9, and anxiety symptoms with the Generalized Anxiety Disorder-7. Red-squared data points and red regression lines (Psych +) represent psychiatrically ill participants (*n* = 25), whereas blue-circled data points and blue regression lines (Psych −) represent psychiatrically healthy ones (*n* = 372). Regression coefficients with their 95% confidence intervals and *p*-values are reported near the regression lines. Comparisons of the regression lines were evaluated by the interaction models and the respective *p*-values were reported. All the presented analyses were adjusted for potential confounders: sex, BMI, physical activity, overall diet quality (pre-exam Starting the Conversation scale), and four out of five personality traits (Big Five Inventory-Short): neuroticism, extraversion, agreeableness, and consciousness.

It has been reported in our previous study that under stress, the consumption of fermented food in psychiatrically healthy medical students was positively associated with the severity of depressive and anxiety symptoms, with a small effect size [17]. These findings were not replicated in the group of psychiatrically ill students. In contrast, the psychiatrically ill students were found to present this association as negative and relatively strong for depressive symptoms: β −0.55 (95% CI −0.91 to −0.19), *p* = 0.0044, but insignificant (although still negative) for anxiety symptoms: β −0.24 (95% CI −0.66 to 0.17), *p* = 0.24, in the raw analyses. Non-parametric analyses resulted in even more negative and significant associations between fermented food consumption and depression as well as anxiety indices in the psychiatrically ill group (Spearman’s ρ −0.63, *p* = 0.0008 for depressive symptoms and Spearman’s ρ −0.42, *p* = 0.036 for anxiety symptoms). GLM analyses adjusted for potential confounders yielded similar results: β −0.52 (95% CI −0.85 to −0.19), *p* = 0.0042 and β −0.22 (95% CI −0.59 to 0.15), *p* = 0.22, for depressive and anxiety symptoms, respectively. A significant interaction was observed between the consumption of fermented food and psychiatric illness in predicting depressive (*p* = 0.0001 and *p* = 0.0001), but not anxiety symptoms (*p* = 0.092 and *p* = 0.053), in both the raw and adjusted analyses, respectively. Detailed results of the GLM interaction models are presented in Table 2 and further illustrated in Figure 1. The models suggest that psychiatrically ill students who consume high amounts of fermented food (>1600 g weekly) tend to present less depressive symptoms under stress than their psychiatrically healthy schoolmates who eat the same amount of fermented food.

### 3.4. Sensitivity and Ancillary Analyses

To validate all these findings, sensitivity analyses were carried out. The analyses included two subgroups of psychiatric participants demonstrating strong adherence to the seven-day dietary recording procedure, i.e., those missing no single day in the food records (*n* = 17) and those declaring to miss no more than 10% of the consumed food records (*n* = 17). Moreover, an analysis was performed with the use of fermented food consumption calculated with the exclusion of probiotic dietary supplements and medicinal products as well as with three-day pre-exam food records in the total sample of psychiatrically ill participants. The sensitivity analyses identified largely significant negative associations for depressive symptoms and were still insignificant for anxiety symptoms, which confirms our main findings. Detailed results of sensitivity analyses are presented in Supplementary Material S2.

Testing each fermented foodstuff and probiotic preparation separately for the association with depressive symptoms in the group of psychiatrically ill participants suggested that all the consumed foods and probiotic preparations contributed to some extent to the reported effect. However, only the consumption of yogurt, kefir, and soured milk returned statistically significant negative relationships with depressive symptoms with the significant interaction between the consumption and psychiatric diagnosis. Further details of this analysis are provided in Supplementary Material S3.

We also tested whether the reported difference in the association between fermented food consumption and depressive symptoms may be explained by baseline mental health rather than psychiatric diagnosis. In a pooled group of psychiatrically ill and healthy participants, we built a GLM model evaluating the interaction between baseline depressive symptoms and fermented food consumption in predicting the outcome of depressive symptoms. The model found weak evidence for the interaction (*p* = 0.091 and *p* = 0.14 for raw and adjusted analysis, respectively) and was insignificant in the psychiatrically healthy group (*p* = 0.37 and *p* = 0.55 for raw and adjusted analysis, respectively), suggesting the association of interest to be negligibly dependent on baseline mental health. Besides, the significant interaction between fermented food consumption and psychiatric diagnosis persisted in a subgroup of students presenting at least moderate baseline depressive symptoms (PHQ-9 ≥ 10 [25]) (*p* = 0.0043 and *p* = 0.0013 for raw and adjusted analysis, respectively—the analysis included 16 psychiatrically ill and 123 healthy students).

## 4. Discussion

Probiotic preparations and fermented foods hold promise for improving mental health [2,3,4,5], as indicated by the results of several systematic reviews of interventional research studies [6,7,8,9,10,11,12,13] and some observational analyses [14,15,16]. Patients suffering from clinical depression appear to benefit from gut microbiota-based therapies or diets more than healthy individuals [8,9,10,11]; however, no research has directly evaluated this relationship. To address this gap, the present study examined the differences in associations between the consumption of fermented food and mental health indices in psychiatrically ill medical students and in their psychiatrically healthy schoolmates. The study found significant differences in the examined associations, suggesting the beneficial effect of fermented food consumption in psychiatric patients, but not in healthy individuals.

The reported findings may be explained by the pathophysiology of mood disorders. Major depression has a strong molecular basis, characterized by excessive oxidative stress and lower antioxidant protection [33,34], resulting in the induction of pro-inflammatory reactions [34,35,36], and in turn, serotonin depletion [37]. The hypothalamic–pituitary–adrenal (HPA) axis is well-known to be disrupted in the course of depression, and some diagnostic and therapeutic strategies targeting HPA have been proposed [38,39]. Depression has also been conceptualized as a neurodevelopmental disease [40], and some neuroplasticity entities such as brain-derived neurotrophic factor (BDNF) deficiency may play a crucial role in its pathophysiology [41]. 

Fermented food, containing potentially probiotic microorganisms [42], may reverse these abnormalities. Probiotics and healthy gut microbes exhibit in vivo antioxidant properties, attenuating oxidative stress [43], present anti-inflammatory properties [43,44,45], may restore serotonergic neurotransmission [45] and normalize the function of the HPA axis [44], and possibly enhance BDNF expression in the brain [46]. All these mechanisms may contribute to the antidepressant action of probiotics [45,47]. Nevertheless, the explanation of the role of fermented food consumption in reversing depressive symptoms should be regarded with caution due to the low sample size of psychiatrically ill participants and correlative character of the present study design.

In contrast to psychiatrically ill individuals, psychiatrically healthy young people may present insignificant biological abnormalities in the above-discussed systems and processes, even under psychological stress—psychiatrically healthy people are simply close to the optimal homeostasis [48]. As a result, there is “nothing to reverse” by probiotics or fermented food in healthy individuals: a phenomenon commonly referred to as a “ceiling effect” [49]. Conversely, while psychiatrically healthy individuals may demonstrate negligible benefits from fermented food, they may also experience detrimental effects from any pathogenic microorganisms or toxins which may be present, as discussed previously [17]. Fermented food may also be both anti-inflammatory and pro-inflammatory, as inflammation is not simply a unidimensional construct [50].

Importantly, the difference in the reported association between the consumption of fermented food and depressive symptoms seems to be explained by psychiatric diagnosis rather than simply baseline mental health symptoms. Mental health symptoms are time-fluctuating in healthy people, being affected by multiple factors such as season [51], trauma [52], or psychological stress [53]. Depressive symptoms experienced under the influence of everyday events in healthy individuals may be reversible and conditioned by only temporal molecular shifts [35]. As a result, there may be “more to reverse” by probiotics or fermented food in psychiatrically ill people than in healthy ones experiencing mental health deterioration with no underlying psychiatric diagnosis.

Fermented food consumption appeared to have a stronger negative association with depression then anxiety symptoms in the psychiatrically ill students. Notably, probiotic interventions have been shown to be more effective in relieving depressive than anxiety symptoms [7,11]. Additionally, the baseline depressive symptoms appeared more exacerbated in psychiatrically ill students than anxiety symptoms, and thus there was “more to reverse” for the depressive symptoms. Nevertheless, our findings do not serve as proof of “no effect” on anxiety, as the statistical power for the performed analyses was not determined and could be insufficient to demonstrate the effect.

The present study has several limitations. First of all, it is an association study, which prevents causative analysis. Nevertheless, the possibility that the extent of fermented food consumption affects mental health status is attractive, mechanistically justifiable, and supported by multiple interventional reports [6,9,10,11,13]. The opposite direction of causation appears less likely, as it is unclear how the same behavioral pattern (consumption of fermented food: yoghurts, cheeses, etc.) would be caused by more anxiety and depression in healthy people, but less anxiety and depression in psychiatric patients. However, this possibility should still be taken into consideration. Secondly, the psychiatric diagnosis of the study participants was self-reported and not based on medical records. However, previous studies have found self-reporting psychiatric diagnosis to have high accuracy [54], with under-reporting being much more frequent than over-reporting [55,56]. As a result, a self-report of suffering from psychiatric disease is likely true in this study, but with some false reports among the psychiatrically healthy group [57]. Additionally, the study used a transparent pre-specified data anonymization procedure [17,18], which secured student privacy and allowed for more honest self-reports. Thirdly, the types of psychiatric diseases the cohort of psychiatrically ill students suffered from have not been examined. However, based on sociodemographic patterns of the participants, general epidemiological data [58,59], and reported mental health symptoms (Table 1), it may be assumed that “psychiatrically ill” largely refers to depressive and anxiety disorders. The group, therefore, may not be very heterogeneous as it comprises socially active medical students, whose sociodemographic characteristics, health-related behaviors, and academic achievements are comparable to those of healthy students. Fourthly, the electronic dietary record method used in the present study was only partially validated. Although it was developed with an external expert, allowed foodstuff mass estimation according to a web-based dietary service, and was pre-tested before the final tool was constructed, the dietary recording method lacks full content, criterion, and construct validation. Finally, the present study is exploratory, it was not set according to any pre-specified research hypothesis, and the reported findings should be interpreted with caution as preliminary.

## 5. Conclusions

Higher consumption of fermented food under stress appears to be associated with less severe depressive symptoms in medical students suffering from a psychiatric condition, with the opposite association noted in psychiatrically healthy medical students. The findings may suggest that psychiatrically ill people, but not healthy ones, benefit from fermented food consumption in terms of alleviation of depressive symptoms. The study is preliminary and requires cautious interpretation. Further investigation with interventional research settings is needed.

## Figures and Tables

**Table 1:** Basal characteristics of the psychiatrically ill medical students compared to those of healthy ones.

Characteristics	Psychiatrically Ill (*n* = 25) ^a^	Psychiatrically Healthy (*n* = 372) ^a^	*p*-Value for Difference
	Mean (Standard Deviation), Median (1st–3rd Quartiles) or Absolute Number (Frequency)		
Faculty			
Faculty of Medicine	19 (76%)	250 (67.2%)	0.36 ^b^
Faculty of Military Medicine	6 (24%)	122 (32.8%)	
Age			
(years)	22.5 (0.7)	22.7 (1.1)	0.79 ^c^
Sex			
Female	19 (76%)	245 (65.9%)	0.30 ^b^
Male	6 (24%)	127 (34.1%)	
Socioeconomic status			
Low	0 (0%)	2 (0.5%)	0.99 ^c^
Middle	18 (72%)	264 (71.0%)	
High	7 (28%)	106 (28.5%)	
Number of inhabitants in a place of family residence			
Below 5000	4 (16%)	88 (23.6%)	0.56 ^c^
5000–50,000	9 (36%)	120 (32.3%)	
50,000–500,000	7 (28%)	91 (24.5%)	
Over 500,000	5 (20%)	73 (19.6%)	
Anthropometry			
Body-mass index (kg × m^−2^)	22.3 (3.3)	22.0 (3.1)	0.80 ^c^
Chronic diseases			
Allergic	6 (24%)	89 (23.9%)	0.99 ^b^
Cardiological	0 (0%)	4 (1.1%)	1.0 ^d^
Endocrine/metabolic	3 (12%)	33 (8.9%)	0.49 ^d^
Gastroenterological	4 (16%)	21 (5.6%)	0.063 ^d^
Immune	0 (0%)	6 (1.6%)	1.0 ^d^
Infectious	0 (0%)	0 (0.0%)	N/A
Neoplastic	0 (0%)	1 (0.3%)	1.0 ^d^
Neurological	1 (4%)	4 (1.1%)	0.28 ^d^
Any chronic disease ^e^	12 (48%)	128 (34.4%)	0.17 ^b^
Personality traits ^f^			
Neuroticism	16 (12–18)	12 (9–15)	0.022 ^c^
Extraversion	12 (8–14)	12 (9–15)	0.95 ^c^
Openness	13 (9–20)	15 (12–17)	0.86 ^c^
Agreeableness	14 (13–16)	15 (12–17)	0.37 ^c^
Conscientiousness	15 (12–19)	16 (14–18)	0.71 ^c^
Health-related behaviors			
Current cigarette smoking/use ^g^	3 (12%)	25 (6.7%)	0.41 ^d^
Physical activity ^h^	3 (2–4)	3 (2–4)	0.36 ^c^
General quality of diet ^i^	8 (6–8)	6 (5–8)	0.40 ^c^
Mode of eating ^j^	6 (5–6)	6 (5–6)	0.77 ^c^
Mental health			
Depressive symptoms ^k^	13 (8–20)	7 (5–11)	<0.0001 ^c^
Anxiety symptoms ^l^	9 (6–15)	6 (4–10)	0.0027 ^c^

N/A—not applicable. ^a^ Substantial difference in the size of the groups reflects the prevalance of psychiatric diseases with possible under-reporting in some participants. ^b^ Pearson’s χ^2^ test. ^c^ Mann–Whitney *U* test. ^d^ Fisher’s exact test. ^e^ Any chronic disease other than psychiatric. ^f^ Expression of five personality traits was presented numerically in the range of 3–21, with the midpoint of 12. Cronbach’s alpha assessed in the study sample of 397 psychiatrically ill and healthy students for the subscales was 0.66 for neuroticism, 0.76 for extraversion, 0.71 for openness, 0.51 for agreeableness, and 0.69 for conscientiousness. ^g^ Fraction of participants reporting either traditional cigarette smoking or e-cigarette use. ^h^ Physical activity was expressed on a 5-point semantic differential scale from 1 (“I have no physical activity at all”) to 5 (“I play sport intensively 5 times a week”), with the midpoint of 3. ^i^ General quality of diet was expressed as the number of points in the Starting the Conversation scale in the range of 0 (maximally healthy diet) to 16 (maximally unhealthy diet), with the midpoint of 8. Cronbach’s alpha assessed in the study sample of 397 psychiatrically ill and healthy students was 0.55. ^j^ Mode of eating was expressed in the 7-point semantic differential scale from 1 (“I only eat products bought in a bar, restaurant, or ready-made products or snacks”) to 7 (“I only eat at home, home-made products”). ^k^ Depressive symptoms were evaluated with the Patient Health Questionnaire-9 scale, range 0–27. Cronbach’s alpha assessed in the study sample of 397 psychiatrically ill and healthy students was 0.82. ^l^ Anxiety symptoms were evaluated with the General Anxiety Disorder-7 scale, range 0-21. Cronbach’s alpha assessed in the study sample of 397 psychiatrically ill and healthy students was 0.89.

**Table 2:** Consumption of fermented food and psychiatric illness interaction models for predicting depressive and anxiety symptoms under psychological stress. The analyses included 25 psychiatrically ill and 372 healthy medical students. The presented analyses were adjusted for potential confounders: sex, BMI, physical activity, overall diet quality (pre-exam Starting the Conversation scale), and four out of five personality traits (Big Five Inventory-Short): neuroticism, extraversion, agreeableness, and consciousness.

Parameter	Slope Factor ^a^		β Regression Coefficient		*p*-Value
	Point Estimate	95% CI	Point Estimate	95% CI	
Prediction of Depressive Symptoms (PHQ-9 score)					
Fermented food consumption	0.9	0.1 to 1.7	0.11	0.01 to 0.20	0.027
Psychiatric illness	8.9	5.8 to 11.9	0.39	0.25 to 0.52	<0.0001
Fermented × Psychiatric	−5.5	−8.2 to −2.8	−0.28	−0.42 to −0.15	0.0001
Prediction of Anxiety Symptoms (GAD-7 score)					
Fermented food consumption	1.0	0.3 to 1.8	0.12	0.03 to 0.21	0.0080
Psychiatric illness	4.8	1.8 to 7.7	0.21	0.08 to 0.34	0.0016
Fermented × Psychiatric	−2.5	−5.1 to 0.0	−0.13	−0.27 to 0.00	0.053

95% CI—95% confidence intervals. PHQ-9—Patient Health Questionnaire-9 scale. Fermented × Psychiatric—interaction between consumption of fermented food and psychiatric illness. GAD-7—Generalized Anxiety Disorder-7 scale. ^a^ Slope factor for each additional 1000 g increase in weekly consumption of fermented food and/or for having a psychiatric disease.

## Data Availability

The data presented in this study are openly available in the Mendeley Data repository at digital object identifier: http://dx.doi.org/10.17632/kdxv4k6f5c.2 accessed on 24 March 2022.

## Supplementary Materials

The following supporting information can be downloaded at: https://www.mdpi.com/article/10.3390/ijerph19073861/s1. Supplementary Material S1: The information provided in the dietary record form to the participants regarding foodstuffs to be classified as fermented. Supplementary Material S2: Association between consumption of fermented food and depressive and anxiety symptoms in psychiatrically ill medical students—sensitivity analyses. Supplementary Material S3: Association between consumption of each fermented foodstuff and depressive symptoms in psychiatrically ill medical students.
