# Supplementary material for: Who Benefits from Fermented Food Consumption? A Comparative Analysis between Psychiatrically Ill and Psychiatrically Healthy Medical Students
Source: Int J Environ Res Public Health. 2022 Mar 24;19(7):3861. doi: 10.3390/ijerph19073861 (PMC8997937; doi:10.3390/ijerph19073861)

Supplementary Material S1

The information provided in the dietary record form to the participants regarding foodstuffs to be classified as fermented

1. **Cheese** (hard cheese – for example gouda, cheddar, parmesan, mozzarella cheese – and soft cheese – for example brie, camembert cheese). Do not include roasted, boiled, fried cheese nor cream cheese.

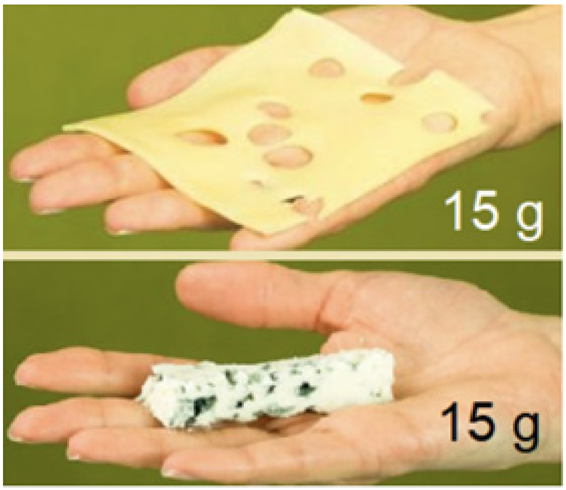

2. **Yogurt, kefir, soured milk** (natural and sweet or flavored, commercial and home-made, whole and low-fat).

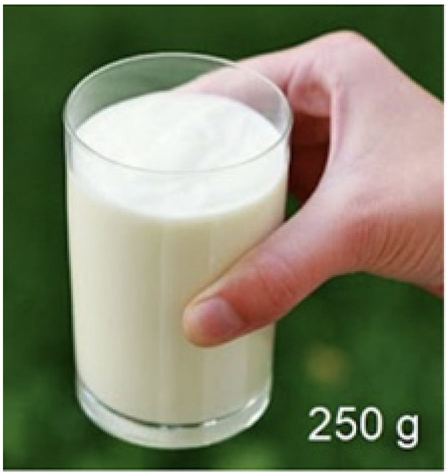

3. **Kvass and unpasteurised beer** (include only products which are unpasteurised, unpreserved, unfiltered and which have short expiry date – unfortunately, the majority of commercial products do not meet these criteria).

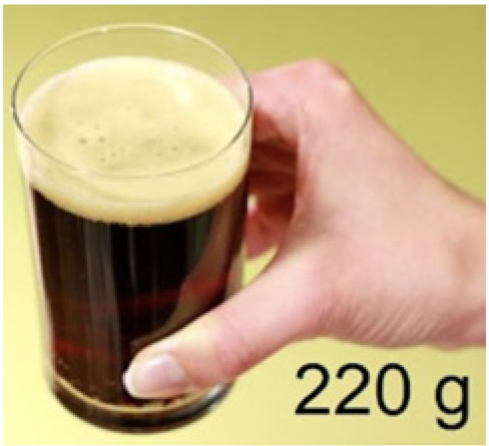

4. **Pickled cucumber and pickling juice** (only raw pickled cucumbers and pickling juice – include neither gherkins nor boiled pickled cucumbers, for example in cucumber soup).

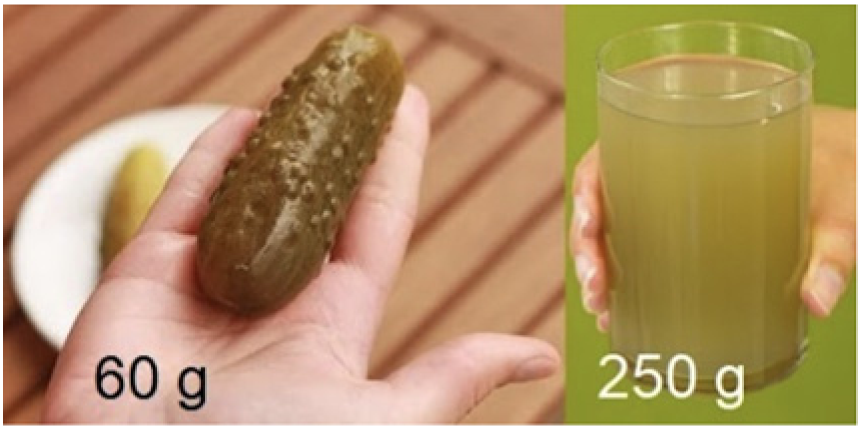

5. **Sauerkraut and pickling juice** (only raw sauerkraut and pickling juice – include neither boiled nor fried sauerkraut).

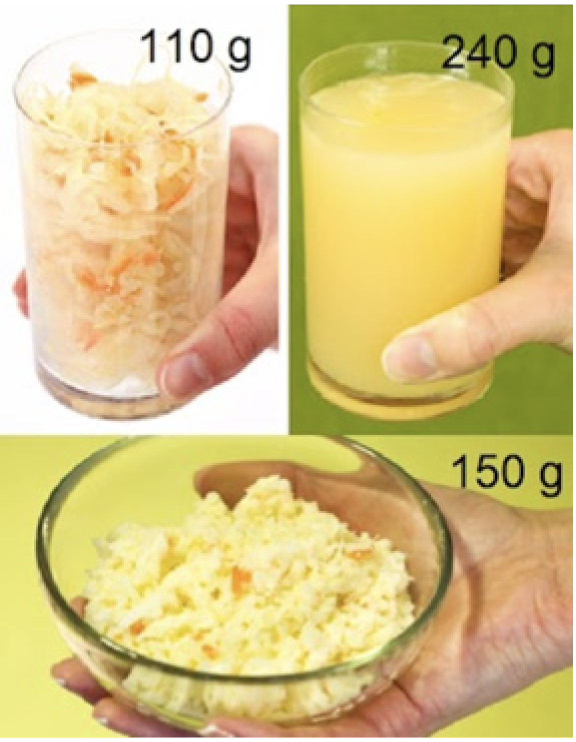

6. **Other fermented vegetables and their pickling juice** (kimchi, fermented beetroots, celeries, broccoli, etc.). Do not include pasteurised products (many commercial products are pasteurized).

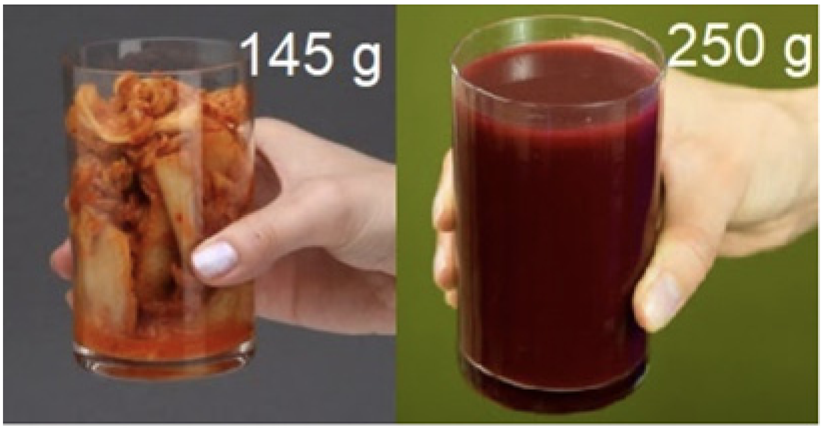

Supplement: Supplementary file 1 [file ijerph-19-03861-s001.zip › Supplementary Material S1.pdf]
