# Supplementary material for: Who Benefits from Fermented Food Consumption? A Comparative Analysis between Psychiatrically Ill and Psychiatrically Healthy Medical Students
Source: Int J Environ Res Public Health. 2022 Mar 24;19(7):3861. doi: 10.3390/ijerph19073861 (PMC8997937; doi:10.3390/ijerph19073861)
Supplement: Supplementary file 1 [file ijerph-19-03861-s001.zip › Supplementary Material S2.pdf]

Supplementary Material S2

Association between consumption of fermented food and depressive and anxiety symptoms in psychiatrically ill medical students – sensitivity analyses

Table S1. Association between consumption of fermented food and **depressive symptoms**

Type of analysis	β (95% CI), *p*-value	
	Raw analysis	Adjusted analysis
A subgroup highly adherent to food recording – missing no single day (n=17)		
Association	-0.50 (-0.98 to -0.02), *p*=0.042	-0.56 (-1.01 to -0.10), *p*=0.023
Interaction	*p*=0.0024	*p*=0.0005
A subgroup highly adherent to food recording – declaring to miss no more than 10% (n=17)		
Association	-0.50 (-0.98 to -0.02), *p*=0.041	-0.49 (-1.01 to 0.02), *p*=0.058
Interaction	*p*=0.0013	*p*=0.0002
Fermented food excluding probiotic dietary supplements and medicinal products (n=25)		
Association	-0.54 (-0.90 to -0.18), *p*=0.0053	-0.52 (-0.87 to -0.17), *p*=0.0064
Interaction	*p*=0.0001	*p*=0.0001
3-day pre-exam food recording (n=25)		
Association	-0.44 (-0.83 to -0.05), *p*=0.028	-0.48 (-0.87 to -0.09), *p*=0.019
Interaction	*p*=0.0023	*p*=0.0019

Table S2. Association between consumption of fermented food and **anxiety symptoms**

Type of analysis	β (95% CI), *p*-value	
	Raw analysis	Adjusted analysis
A subgroup highly adherent to food recording – missing no single day (n=17)		
Association	0.07 (-0.48 to 0.62), *p*=0.79	-0.03 (-0.51 to 0.45), *p*=0.88
Interaction	*p*=0.61	*p*=0.37
A subgroup highly adherent to food recording – declaring to miss no more than 10% (n=17)		
Association	-0.22 (-0.76 to 0.31), *p*=0.39	-0.21 (-0.74 to 0.32), *p*=0.38
Interaction	*p*=0.20	*p*=0.088
Fermented food excluding probiotic dietary supplements and medicinal products (n=25)		
Association	-0.27 (-0.68 to 0.15), *p*=0.20	-0.19 (-0.58 to 0.20), *p*=0.31
Interaction	*p*=0.061	*p*=0.065
3-day pre-exam food recording (n=25)		
Association	-0.25 (-0.67 to 0.16), *p*=0.22	-0.20 (-0.60 to 0.20), *p*=0.32
Interaction	*p*=0.073	*p*=0.054
