# Supplementary material for: Who Benefits from Fermented Food Consumption? A Comparative Analysis between Psychiatrically Ill and Psychiatrically Healthy Medical Students
Source: Int J Environ Res Public Health. 2022 Mar 24;19(7):3861. doi: 10.3390/ijerph19073861 (PMC8997937; doi:10.3390/ijerph19073861)
Supplement: Supplementary file 1 [file ijerph-19-03861-s001.zip › Supplementary Material S3.pdf]

Supplementary Material S3

Association between consumption of each fermented foodstuff and depressive symptoms in psychiatrically ill medical students

Table S3. Association between consumption of each fermented foodstuff and **depressive symptoms**

Type of analysis	β (95% CI), *p*-value	
	Raw analysis	Adjusted analysis
**Cheese** [a]		
Association	-0.23 (-0.65 to 0.19), *p*=0.26	-0.25 (-0.69 to 0.20), *p*=0.25
Interaction	*p*=0.042	*p*=0.037
**Yogurt, kefir and soured milk** [a]		
Association	-0.46 (-0.85 to -0.08), *p*=0.020	-0.49 (-0.89 to -0.08), *p*=0.023
Interaction	*p*=0.0026	*p*=0.0046
**Pickled cucumber and pickling juice** [a]		
Association	-0.19 (-0.61 to 0.24), *p*=0.37	-0.27 (-0.71 to 0.18), *p*=0.23
Interaction	*p*=0.28	*p*=0.12
**Probiotic dietary supplements and medicinal products** [a]		
Association	-0.19 (-0.61 to 0.23), *p*=0.36	-0.30 (-0.72 to 0.12), *p*=0.15
Interaction	*p*=0.93	*p*=0.57

[a] Other fermented foodstuffs were not reported to be consumed by the psychiatrically-ill in the 7-day period of food recording.
